# Comparative efficacy of r-hFSH Alfa + r-LH versus r-hFSH delta + hMG in poor ovarian responders

**DOI:** 10.3389/fendo.2025.1656569

**Published:** 2025-11-25

**Authors:** Giorgio Maria Baldini, Domenico Baldini, Dario Lot, Antonio Malvasi, Antonio Simone Laganà, Mario Palumbo, Gianluca Raffaello Damiani, Giuseppe Trojano

**Affiliations:** 1 Obstetrics and Gynecology Unit, Department of Biomedical Sciences and Human Oncology, University of Bari “Aldo Moro”, Bari, Italy; 2 IVF Center, Momo Fertilife, Bisceglie, Italy; 3 Unit of Obstetrics and Gynecology, “Paolo Giaccone” Hospital, Department of Health Promotion, Mother and Childcare, Internal Medicine and Medical Specialties (PROMISE), University of Palermo, Palermo, Italy; 4 Department of Public Health, School of Medicine, University of Naples “Federico II”, Naples, Italy; 5 Department of Maternal and Child Health, Madonna Delle Grazie Hospital, Matera, Italy

**Keywords:** rFSH, RLH, poor responder, stimulation protocol, controlled ovarian stimulation

## Abstract

**Introduction:**

Poor ovarian response (POR) remains a major limitation in assisted reproductive medicine. Direct comparisons between r-hFSH alfa + r-LH and r-hFSH delta + hMG in this population are scarce.

**Methods:**

This retrospective study included 437 POR patients treated between 2018 and 2022. Controlled ovarian stimulation involved either r-hFSH alfa + r-LH (Group A) or r-hFSH delta + hMG (Group B). After exclusions, 148 patients per group who reached embryo transfer were analyzed. Primary outcomes were total oocytes, metaphase II (MII) oocytes and embryos. Secondary outcomes included β-hCG positivity, clinical pregnancy and pharmacoeconomic evaluation.

**Results:**

Group A showed significantly higher numbers of oocytes (p=0.01), MII oocytes (p=0.02) and embryos (p=0.03). No significant differences in biochemical or clinical pregnancy were observed (OR 1.12; 95% CI: 0.79–1.59). The r-hFSH alfa + r-LH protocol required lower total gonadotropin doses and resulted in an average saving of €690 per additional MII oocyte.

**Discussion:**

The r-hFSH alfa + r-LH regimen improved ovarian response and cost-efficiency without affecting pregnancy outcomes. Prospective randomized trials are needed to confirm these results.

## 1 Introduction

Ovarian stimulation in poor responders remains one of the most challenging aspects of assisted reproductive medicine, despite the multiple strategies proposed and the numerous studies conducted (1). This subset of patients is characterized by a limited number of oocytes retrieved and significantly lower pregnancy rates compared to the general population (2), prompting ongoing efforts within the scientific community to investigate and develop alternative therapeutic approaches (3–5). Among these, the use of recombinant gonadotropins, such as follitropin alfa and delta, has raised interest due to the greater uniformity of response compared to urinary formulations (4). Furthermore, supplementation with recombinant human luteinizing hormone (LH) or human menopausal gonadotropin (HMG), such as Meropur, has been proposed to promote a more effective follicular maturation, compensating for the reduced endogenous LH secretion in this category of patients (6, 7). The combination of recombinant human Follicle Stimulatin Hormone (FSH) with HMG has been explored in preliminary studies, suggesting a potential improvement in follicular response and clinical outcomes; however, the evidence remains limited (6, 8). However, a direct comparison between two specific therapeutic regimens is still missing: follitropin alfa + recombinant LH (Pergoveris) and follitropin delta + HMG (Rekovelle + Meropur). The starting hypothesis of this study is that the combination of follitropin alfa and recombinant LH may offer superior ovarian response and be more cost-effective than the protocol using follitropin delta and HMG while maintaining similar pregnancy rates. The study, therefore, aims to compare the two protocols in terms of oocytes retrieved, metaphase II oocytes, and embryos produced, as well as secondary outcomes, including β-hCG positivity and clinical pregnancy rate and cost-efficiency.

## 2 Materials and methods

### 2.1 Patient selection

**Figure 1:**
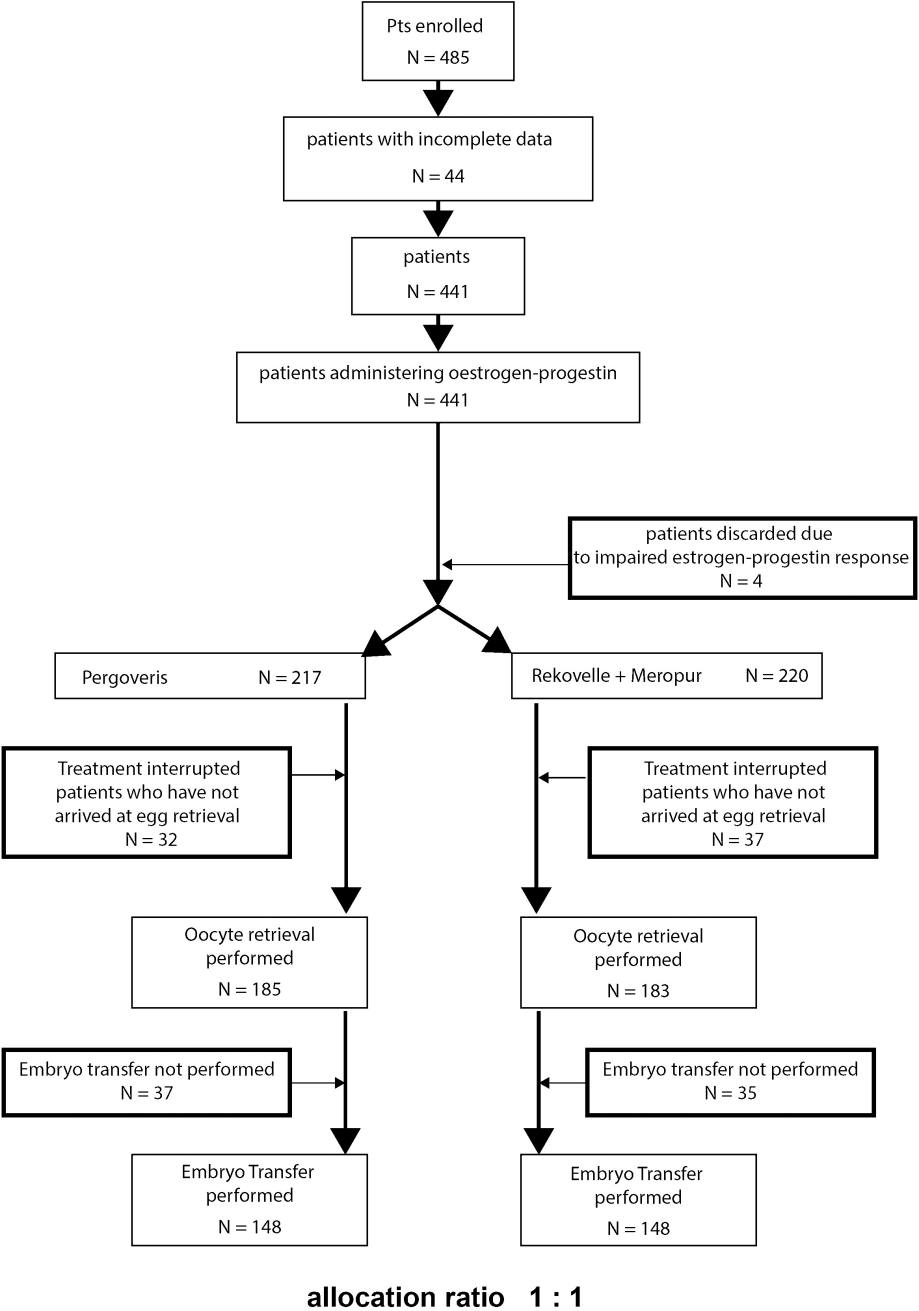
Draw of study.

Between January 2018 and October 2022, approximately 485 patients with a diagnosis of poor ovarian response were treated at the private medically assisted procreation centre MOMÒ Fertilife in Bisceglie (BT) (9). The inclusion criteria included the diagnosis of poor ovarian response according to the Bologna criteria (9), reduced Anti-Müllerian Hormone (AMH) values (<1.0 ng/mL) and/or antral follicle count (AFC =< 5-6), or an insufficient response to stimulation in the presence of normal ovarian reserve parameters (10). Poor ovarian response was defined according to the ESHRE consensus established in Bologna in 2011, which identifies this condition when at least two of the following are present: advanced maternal age of 40 years or older or other risk factors for reduced ovarian reserve, a previous episode of poor ovarian response defined as the retrieval of three or fewer oocytes after standard stimulation, and abnormal ovarian reserve parameters such as a low antral follicle count (below 5–7) or reduced serum anti-Müllerian hormone (AMH) levels (below 0.5–1.1 ng/mL). These criteria provide a standardized framework for identifying women with a limited quantitative ovarian response, although they may not fully capture the heterogeneity and clinical complexity of this patient population. Patients with severe male factor problems (cryptozoospermia, severe OATS), as well as those with male or female genetic anomalies, were excluded from the study as seen in **Figure 1**. Forty-four cases were excluded due to incomplete data, and four additional patients discontinued treatment during the preparatory phase with estrogen–progestin therapy. A total of 437 patients were therefore included, with 217 allocated to Group A (rFSH alfa + rLH; Pergoveris, Merck Serono) and 220 to Group B (rFSH delta + hMG; Rekovelle + Meropur, Ferring). During stimulation, oocyte retrieval was not achieved in 32 patients from Group A (14.7%) and 37 from Group B (16.8%). Among those who underwent oocyte pick-up, 148 patients in each group proceeded to embryo transfer, while the remaining cases did not obtain transferable embryos. For the final comparative analysis, only patients who reached embryo transfer were included to ensure sample homogeneity and minimize potential statistical bias.

### 2.2 Study design

The study was designed as an observational cross sectional. The study protocol was approved by the local ethics committee (CELMF n° 12/2018). The study was conducted by the International Conference on Harmonisation of Guidelines, as outlined in the Helsinki Declaration. All couples signed an informed consent to participate in the study.

### 2.3 Ovarian stimulation and oocyte retrieval

All patients underwent a standardized protocol that included the administration of combined estrogen–progestin therapy in preparation for the onset of menstruation. Group A was treated with a fixed dose of 300 IU of Pergoveris (Pergoveris, Merck Serono, Frankfurter Straße250, 64293 Darmstadt, Germany) starting from the second day of menstruation. The daily administration ratio included 300 IU of follitropin alfa and 150 IU of LH. Group B started on the second day of the cycle with a fixed dose of 10 mcg Rekovelle (Ferring Pharmaceuticals, SaintPrex, Switzerland) (approximately equivalent to 150 IU rFSH delta) (11) in addition to 1 vial of Meropur (HMG) (Ferring Pharmaceuticals, Chemin de la Vergognausaz 50, 1162 SaintPrex, Switzerland) containing 150 IU FSH and 150 IU LH/hCG activity, thus obtaining a total daily dosage of approximately 300 IU FSH and 150 IU LH/hCG activity. Meropur was used to provide both an additional FSH and the necessary LH activity, mainly mediated by hCG. Although Rekovelle is approved for individualized dosing based on AMH and BMI, a fixed daily dose of 10 micrograms was used in this study to ensure strict protocol standardisation and minimise variability among patients. This methodological choice may limit the generalizability of the results and should be taken into account when interpreting the findings (12). In patients with poor responder status and very low AMH, the algorithm-based Rekovelle dose typically results in the maximum recommended range (10–12 mcg). Therefore, a fixed dose of 10 mcg was selected as representative for this population. On day 8 of the cycle, both groups initiated the antagonist; in group A, Cetrotide (Cetrotide, Merck Serono, Feltham, UK) was administered at 0.25 daily; in group B, Fyremadel (Fyremadel, Ferring B.V., Hoofddorp, The Netherlands) was administered at 0.25 daily. Daily administration of gonadotropins continued until at least one or two follicles reached a diameter >= 18 mm. Monitoring was performed with transvaginal ultrasound and evaluation of serum E2 and Progesterone levels. In any case, stimulation did not exceed 18 days. The trigger was obtained with the administration of a dose of 10,000 IU s.c. of hCG (Gonasi-HP, IBSA Italia, Lodi, Italy). Oocyte collection was performed 34–37 hours after the trigger. The oocytes were then fertilised using ICSI which is the standard insemination method routinely performed to ensure procedural uniformity and minimize fertilization variability across cases, according to the standardised protocol employed in our laboratory (13, 14). Embryo transfer was performed after two or three days. Luteal phase support was performed using progesterone (Amelgen; Gedeon Richter Plc. Gyomroi ut 19-21 1103, Budapest, Hungary) administered vaginally at a dose of 400 mg x 2/day (15). Twelve days after oocyte retrieval, the patient performed a blood pregnancy test. Subsequently, a transvaginal ultrasound was performed to evaluate whether there was clinical evolution of the pregnancy.

### 2.4 Outcome measure

The primary endpoints were the number of oocytes retrieved, the number of metaphase II oocytes, and the number of embryos transferred. The beta hCG test and clinical pregnancy were the secondary endpoints. Clinical pregnancy was defined as the presence of a fetal heartbeat. Finally, a pharmacoeconomic evaluation was conducted to compare the cost associated with only FSH and LH-based drugs used in the two treatment protocols.

### 2.5 Sample size calculation and statistical methods

Given the retrospective nature of this study, no *a priori* sample size calculation was performed. Instead, all eligible cases within the study period were included. To minimize selection bias and balance key baseline characteristics, propensity score matching (PSM) was performed using a logistic regression model that included age, AMH, AFC, and BMI as covariates. Matching was conducted using a nearest-neighbour algorithm without replacement at a 1:1 ratio and a caliper width of 0.2 standard deviations of the logit of the propensity score. The PSM analysis was conducted using [SPSS version (5)]. Patients without a suitable match were excluded from the final comparative analysis. The balance of covariates before and after matching was assessed using standardized mean differences (SMD) and presented in a supplementary table and flow diagram. Descriptive statistics were calculated, and comparative analyses were conducted using Student’s t- test or Mann-Whitney test as appropriate. Categorical variables were compared with Fisher’s exact test or Chi-square test. Statistical significance was defined as a two-tailed p-value <0.05. Analyzes were performed with Prism (GraphPad Software, Boston, MA, USA).

## 3 Results

Between January 2018 and October 2022 (the date Ferring suspended the marketing of Meropur in Italy), 485 couples with a female diagnosis of poor responder were treated. Of these 485 couples, 44 did not have complete data and were excluded, while 441 began cycle preparation with estrogen progestins. During the administration of estrogen progestins, four patients had irregularities and, therefore, abandoned the protocol. Finally, 437 patients were selected to participate in the study, of which 217 were in group A (rFSH alpha + rLH, Pergoveris Merck Serono) and 220 were in group B (rFSH delta + HMG Rekovelle + Meropur Ferring). During stimulation, 32 patients in group A (14.7%) and 37 patients in group B (16.8%) did not reach oocyte collection. Therefore, 185 and 183 oocyte collections were performed, respectively. In group A, only 148 patients reached transfer; 37 (20%) did not have transferable embryos. In group B, 148 patients reached transfer, and 35 (17.11%) did not have transferable embryos. The dropout rates of the two groups were similar to those found in the scientific literature (1, 3, 16–25). For the final comparative analysis, patients who reached embryo transfer (ET) were selected. This approach was adopted to maintain homogeneity in the sample size and minimize the risk of statistical bias. The 148 patients in each group were then compared according to epidemiological and anamnestic variables (age, previous treatments, duration of infertility, BMI, and indications for treatment), without statistically significant differences being highlighted using the Student t-test (**Table 1**). Hormonal and ovarian reserve parameters (basal FSH, basal E2, AFC, AMH) were evaluated with the Mann-Whitney test since the Shapiro test did not confirm a normal distribution. Also, in this case, no statistically significant differences emerged as showed in **Table 1**.

**Table 1:** Characteristic epidemiologic and basal situation between the two study groups.

Patients with embryo transfer	Group A		Group B		CI	*p-value*
	rFSH alfa+rLH		rFSH delta+ HMG			
	Pergoveris		Rekovelle + Meropur			
	148		148			
	Media	SD	Media	SD		
Age	38,402	3,7427	38,994	4,2805	-0,2141 to 1,400	0.1494*
Previous treatment	1,8571	1,0135	1,801	1,04572	-0,2626 to 0,1504	0.5933*
Infertility duration	3,8717	2,4262	3,901	2,2757	-0,4921 to 0,4630	0.9524*
BMI	23,29153	3,3874	23,28	4,6672	-0,8337 to 0,8057	0.9733*
Indication						
Male factor	25	16,9%	22	14,9%	–	0.6852*
Male factor + female factor	35	23,6%	32	21,6%	–	0.1863*
Endometriosis	22	14,9%	24	16,2%	–	0.1746*
Tubal abnormalities	6	4,1%	8	5,4%	–	0.5482*
POR	27	18,2%	26	17,6%	–	0.8329*
Other	33	22,3%	36	24,3%	–	0.4885*
Total	148		148			
FSH basal (IU/L)	10,28	4,93	10,353	4,443	–	0,3627**
E2 basal (pg/mL)	69,76	39,55	70,3383	50,9014	–	0,3382**
AMH (ng/mL)	0,9	0,55	0,8958	0,7206	–	0,2030**
AFC (n)	6,44	2,7	6,5102	2,4061	–	0,4770**

*Student’s *t*-test; **Mann–Whitney U test; SD, standard deviation; CI, Confidence Interval.

In **Table 2**, the parameters regarding the response to stimulation were evaluated. The duration of stimulation, the number of follicles present and the day of transfer were not significant. However, E2 and Progesterone on the day of the trigger were statistically significant in favour of the group that administered Pergoveris. The number of oocytes retrieved and the number of oocytes in metaphase II were also statistically significant in favour of the group that administered Pergoveris, especially the oocytes in metaphase II. We performed multivariate logistic regression to evaluate the association between treatment and the probability of obtaining ≥3 oocytes in metaphase II, controlling for age, AMH, AFC and BMI. Treatment with Pergoveris showed a favourable trend (OR = 1.51, p = 0.067), although not statistically significant. AMH (p = 0.004) and AFC (p = 0.045) were found to be significant independent predictors of ovarian response.

**Table 2:** Comparison of responses to stimulation between the two study groups.

Patients with embryo transfer	Group A		Group B		*p-value*
	rFSH alfa+rLH		rFSH delta+ HMG		
	(Pergoveris)		Rekovelle + Meropur		
	n = 148		148		
	Mean	SD	Media	SD	
Stimulation duration	11,14	1,93	11,2857	1,9215	0,9306*
E2 trigger day	**1069,76**	**390,55**	961,8547	707,2243	0,0151*
Progesterone trigger day	1,01	0,65	0,8491	0,89412	0,0002*
FSH from Rekovelle in mcg	—	—	94,53	12,27	—
conversion in IU	—	—	1418	0.85	—
FSH from Meropur in IU	—	—	1435	705	—
total FSH in IU	2678	1390	2853	1423	0.0678*
Total LH in IU	1339	695	1435	705	0.2752*
Complication	0	—	0	—	—
N°Follicles	6,67	3,24	6,2857	3,7347	0,3305*
Oocytes retrieval	4,3	2,13	3,8163	2,3592	0,0261*
Oocytes MII	2,714	1,384	2,571	1,556	<0,0001*
N° embryo transfer	1,39	0,93	1,1836	0,775879	0,0301*
Day transfer	2,492	1,528	2,4081	1,4594	0,9988*

Statistical significance (p < 0.05) indicated in bold.

Mann–Whitney U test used. IU, International Unit; MII, metaphase II; SD, standard deviation.

To evaluate the outcomes of positive beta-hCG and clinical pregnancy in the Rekovelle + Meropur group compared to the Pergoveris group, we used Fisher’s exact test and calculated the Odds Ratio (OR). Both statistical approaches indicated no significant differences between the two treatment groups. Specifically, all Fisher’s exact tests resulted in non-significant results, and although the Odds Ratios were greater than 1, they did not reach statistical significance (see **Table 3**). Similarly, the data on miscarriage rates within the first 12 weeks of pregnancy also showed no statistically significant differences between the groups.

**Table 3:** Evaluation of betaHcg and clinical pregnancy rates between the two study groups.

Patients with embryo transfer	Group A		Group B		Test	Test	95% CI
	rFSH alfa+rLH		rFSH delta+ HMG				
	Pergoveris		Rekovelle + Meropur		Fischer’s exact	Odds ratio	
	148		148				
Beta hcg positive	25	16,80%	22	14,86%	0,7507	**1,164**	0,6311 to 2,189
Clinical Pregnancy	18	12,16%	17	11,48	>0,9999	**1,067**	0,5124 to 2,087
Miscarriage within the 12th week	3	16,66%	3	17,6%	0,8904	**1,098**	0,4378 to 2,153

C.I., Confidence Interval.

Bold values indicate statistical significance (p < 0.05).

A multivariate logistic regression analysis was performed to assess the independent effect of protocol type on the clinical pregnancy rate. The model included age, AMH, AFC, BMI, duration of infertility, and number of previous treatments as covariates. Treatment with Pergoveris was associated with a non-significant increase in the probability of clinical pregnancy (OR = 1.18; 95% CI: 0.63–2.21; p = 0.59). The pharmacoeconomic analysis comparing the two protocols used, based on ex-factory prices net of legal reductions (Pergoveris: Determination 15 September 2020, no. DG/941/2020 – GU General Series no. 238 of 25-09-2020; Rekovelle: Determination 21 February 2020, no. 215/2020 – GU General Series no. 54 of 03-03-2020; Meropur: Determination 7 April 2006, no. 210/2006 -GU General Series no. 91 of 19/04/2006) highlights the following. In the Pergoveris protocol, a total of 2,678 IU of FSH was used per stimulation cycle for a total cost of €1,010.58. Conversely, the Rekovelle+Meropur regimen involved the use of 2,853 IU in total (1,418 IU Rekovelle and 1,435 IU Meropur), with a total cost of €1,109.21, indicating a cost increase of approximately 9.76% and an incremental use of 175 IU. The ICER (incremental cost per additional IU) calculated on these values is approximately €0.56 for each IU. Although the primary endpoint did not demonstrate any difference, evaluating the efficacy in terms of oocytes retrieved, the Pergoveris protocol allows, on average, the recovery of 2,714 MII oocytes against 2,571 obtained with the Rekovelle+Meropur combination, with a difference of 0.143 more oocytes in favour of Pergoveris.

## 4 Discussion

Despite several studies conducted (1, 18), no solution has emerged that can significantly improve success rates for poor responders. Recombinant gonadotropins such as follitropin alfa and delta could offer advantages in terms of efficacy and safety (5). This scenario has prompted the scientific community to explore modified protocols and the addition of hormones, such as LH or HMG (3, 5, 26). Furthermore, studies have suggested that LH supplementation may help improve the quality of ovarian response in poor responders (5, 6, 26, 27). Recent studies have shown that the use of follitropin delta can lead to implantation and pregnancy rates similar to those obtained with conventional FSH (28). A meta-analysis of gonadotropin combinations showed a higher pregnancy rate in patients treated with a combination of r-hFSH and r-hLH compared to those treated with hMG or FSH alone (29–33). The efficacy of r-hLH, compared to hMG, could be linked to its ability to stimulate LH receptors in a more targeted way, as highlighted by Dahan et al. (30).

Furthermore, LH and hCG, although activating the same receptor (LHCGR), evoke different cellular and molecular responses (33). Our study compared the clinical results obtained with two different gonadotropin regimens, r-hFSH delta + hMG versus r-hFSH alpha + r-hLH, during controlled ovarian stimulation (COS) treatment in women with poor ovarian response. This study is the first to explore such associations. Our study reported different and contrasting results with those of Bissonnette et al. (34), who showed an improvement in both the number and quality of blastocysts with the association of follitropin delta and menotropin. However, it is essential to note that the study by Bissonnette et al. (35) was conducted on a general population rather than specifically on poor responders, a characteristic that may explain the differences observed in the results. We used a fixed dose to minimize variations that could influence the results, an approach also supported by the Cochrane Library, which suggested that fixed doses of gonadotropins (300–450 IU of FSH) lead to an improvement in the number of oocytes in poor responders (36–38). Although PSM was used to balance baseline characteristics, residual confounding cannot be fully excluded. Additionally, matching reduced the sample size, which may affect statistical power. In our study, the r-hFSH alpha + r-hLH group showed a significantly higher increase in E2 levels on the trigger day. Progesterone on the trigger day was also statistically significant in favour of the r- hFSH alpha + r-hLH group. However, since this value always remained below 1.5 ng/mL, we believe it did not impact the treatment outcome in any way. The number of oocytes retrieved, the number of mature oocytes and the number of embryos transferred were also statistically significant in favour of Group A. However, despite the higher number of oocytes retrieved, the pregnancy rate did not show statistically significant differences between the two groups (39, 40). In both cases, multivariate logistic regression did not identify statistically significant differences. Both treatment groups were found to be safe, without complications, in line with what was reported by Kovacs et al. (41) and Humaidan et al. (42). Statistical tests on beta-hCG and clinical pregnancy outcomes showed that both regimens produced similar results, as evidenced in several comparative studies similar to ours (43–46). The pharmacoeconomic evaluation highlighted an incremental cost difference, equal to –€98.63 (a negative value indicating that Pergoveris is less expensive), leading to an ICER of approximately –€689.6 for each additional MII oocyte. This result confirms that the Pergoveris protocol is certainly more economical, offering a saving of approximately €690 for each additional MII oocyte retrieved. A recent study by Meyer et al. (47) compared the total costs of an ovarian stimulation cycle using follitropin alfa versus urinary follitropin with the addition of LH or HMG. The results showed that, despite the initially higher cost of recombinant gonadotropins, the use of lower doses and the potential reduction in the number of cycles required for oocyte retrieval resulted in an overall decrease in long-term costs (47).

### 4.1 Limitation of the study

The study, due to its retrospective nature, is susceptible to selection bias and residual confounding despite the apparent homogeneity of the groups. The adoption of the Bologna criteria, although the current standard, does not always reflect the clinical complexity of the “poor responder” phenotype and could introduce heterogeneity in the sample (48, 49). Furthermore, the difference between the recombinant gonadotropins used makes it difficult to isolate the effect of r-hFSH compared to that of the r-hLH and HMG combinations. Although Rekovelle is approved for individualised dosing based on AMH and BMI, this study employed a fixed daily dose of 10 mcg to ensure strict protocol standardisation and minimise variability among patients. This methodological choice may limit the generalizability of the results and should be taken into account when interpreting the findings. Furthermore, since the study population consisted of women with poor ovarian response, asynchronous follicular growth represents an additional biological concern. This phenomenon, characterized by variability in follicular development and size within a numerically limited cohort, may lead to a lower yield of mature, developmentally competent oocytes, a reduced number of embryos available for transfer, and consequently a diminished likelihood of achieving pregnancy (50, 51). As previously reported, individualized follitropin delta dosing according to body weight and AMH can mitigate this risk in some populations (52). Therefore, the absence of individualized dosing in our protocol should be acknowledged as an additional limitation that may have influenced the ovarian response in this study. The study is also monocentric, limiting the generalizability of the results.

## 5 Conclusions

Our study showed a significantly higher number of mature oocytes in the group treated with r-hFSH alpha + r-LH, suggesting a possible greater efficiency of this combination compared to the regimen with r-hFSH delta + hMG. However, multivariate analysis did not confirm a significant clinical impact, and pregnancy rates remained similar between groups. From an economic point of view, the Pergoveris protocol showed an advantage in both terms of cost per cycle and cost per MII oocyte retrieved, resulting in a more sustainable approach. These results, although promising, do not change the clinical challenge represented by poor responder patients, for whom pregnancy rates remain unsatisfactory. Therefore, prospective, randomised, and multicenter studies with a large sample of patients will be necessary to confirm and validate these findings. Such studies could provide more solid evidence to guide the optimization of therapeutic protocols, thereby improving the clinical management of this challenging patient population.

## Data Availability

The raw data supporting the conclusions of this article will be made available by the authors, without undue reservation.
