# Correction: Comparative efficacy of r-hFSH Alfa + r-LH versus r-hFSH delta + hMG in poor ovarian responders

**DOI:** 10.3389/fendo.2026.1813345

**Published:** 2026-03-06

**Authors:** Giorgio Maria Baldini, Domenico Baldini, Dario Lot, Antonio Malvasi, Antonio Simone Laganà, Mario Palumbo, Gianluca Raffaello Damiani, Giuseppe Trojano

**Affiliations:** 1 Obstetrics and Gynecology Unit, Department of Biomedical Sciences and Human Oncology, University of Bari “Aldo Moro”, Bari, Italy; 2 IVF Center, Momo Fertilife, Bisceglie, Italy; 3 Unit of Obstetrics and Gynecology, “Paolo Giaccone” Hospital, Department of Health Promotion, Mother and Childcare, Internal Medicine and Medical Specialties (PROMISE), University of Palermo, Palermo, Italy; 4 Department of Public Health, School of Medicine, University of Naples “Federico II”, Naples, Italy; 5 Department of Maternal and Child Health, Madonna Delle Grazie Hospital, Matera, Italy

**Keywords:** rFSH, RLH, poor responder, stimulation protocol, controlled ovarian stimulation

There was a mistake in **Table 2** as published. Specifically, in the row “E2 trigger day”, the mean and SD values for Group A were incorrectly reported as 69,76 and 39,55; the correct values are 1069,76 and 390,55.

**Table 2:** Comparison of responses to stimulation between the two study groups.

Patients with embryo transfer	Group A *rFSH alfa + rLH (Pergoveris)* n = 148	SD	Group B *rFSH delta + HMG (Rekovelle + Meropur)* n = 148	SD	p-value
	Mean		Mean		
Stimulation duration	11,14	1,93	11,2857	1,9215	0,9306*
E2 trigger day	**1069,76**	**390,55**	961,8547	707,2243	0,0151*
Progesterone trigger day	1,01	0,65	0,8491	0,89412	0,0002*
FSH from Rekovelle in mcg	—	—	94,53	12,27	—
Conversion in IU	—	—	1418	0,85	—
FSH from Meropur in IU	—	—	1435	705	—
Total FSH in IU	2678	1390	2853	1423	0,0678*
Total LH in IU	1339	695	1435	705	0,2752*
Complication	0	—	0	—	—
N°Follicles	6,67	3,24	6,2857	3,7347	0,3055*
Oocytes retrieval	4,3	2,13	3,8163	2,3592	0,0261*
Oocytes MII	2,714	1,384	2,571	1,556	<0,0001*
N° embryo transfer	1,39	0,93	1,1836	0,775879	0,0301*
Day transfer	2,492	1,528	2,4081	1,4594	0,9988*

Statistical significance (p < 0.05) indicated in bold.

Mann–Whitney U test used. IU, International Unit; MII, metaphase II; SD, standard deviation.

The corrected **Table 2** appears below.

The original version of this article has been updated.

